# Comparative Transcriptome Analysis of Genes Involved in GA-GID1-DELLA Regulatory Module in Symbiotic and Asymbiotic Seed Germination of *Anoectochilus roxburghii* (Wall.) Lindl. (Orchidaceae)

**DOI:** 10.3390/ijms161226224

**Published:** 2015-12-18

**Authors:** Si-Si Liu, Juan Chen, Shu-Chao Li, Xu Zeng, Zhi-Xia Meng, Shun-Xing Guo

**Affiliations:** Institute of Medicinal Plant Development, Chinese Academy of Medical Sciences & Peking Union Medical College, Beijing 100193, China; liusisi274@126.com (S.-S.L.); kibchenjuan@126.com (J.C.); lixiaofei1117@126.com (S.-C.L.); zengxu1986@sina.com (X.Z.)

**Keywords:** Orchidaceae, *Anoectochilus roxburghii*, transcriptome, seed germination, symbiotic, asymbiotic, GA-GID1-DELLA

## Abstract

*Anoectochilus roxburghii* (Wall.) Lindl. (Orchidaceae) is an endangered medicinal plant in China, also called “King Medicine”. Due to lacking of sufficient nutrients in dust-like seeds, orchid species depend on mycorrhizal fungi for seed germination in the wild. As part of a conservation plan for the species, research on seed germination is necessary. However, the molecular mechanism of seed germination and underlying orchid-fungus interactions during symbiotic germination are poorly understood. In this study, Illumina HiSeq 4000 transcriptome sequencing was performed to generate a substantial sequence dataset of germinating *A. roxburghii* seed. A mean of 44,214,845 clean reads were obtained from each sample. 173,781 unigenes with a mean length of 653 nt were obtained. A total of 51,514 (29.64%) sequences were annotated, among these, 49 unigenes encoding proteins involved in GA-GID1-DELLA regulatory module, including 31 unigenes involved in GA metabolism pathway, 5 unigenes encoding GID1, 11 unigenes for DELLA and 2 unigenes for GID2. A total of 11,881 genes showed significant differential expression in the symbiotic germinating seed sample compared with the asymbiotic germinating seed sample, of which six were involved in the GA-GID1-DELLA regulatory module, and suggested that they might be induced or suppressed by fungi. These results will help us understand better the molecular mechanism of orchid seed germination and orchid-fungus symbiosis.

## 1. Introduction

Bioactive gibberellins (GAs) are diterpene phytohormones that modulate plant growth and development processes, including seed germination, stem elongation, leaf expansion, and reproductive development [1,2]. GA is perceived by GIBBERELLIN INSENSITIVE DWARF1 (GID1) receptors, the GA-GID1 complex is able to interact with DELLA proteins (which are negative regulators of the GA action) when binding to the receptor. The formation of the GA-GID1-DELLA complex triggers enhanced recognition of DELLA by the F-box protein SLY1/GID2, that is part of the SCF^SLY1/GID2^ E3 ubiquitin ligase complex, targets the DELLA repressors for degradation via ubiquitin/26S proteasome [3,4]. The GA-GID1-DELLA signaling module regulates plant growth and development by integrating multiple environmental cues and endogenous signals [5,6].

GA biosynthesis and catabolism pathway in plants is well defined, and genes encoding most enzymes in this pathway have been identified, GAs are biosynthesized from precursor *trans*-geranylgeranyl diphosphate (GGPP) catalyzed by three types of enzymes, terpene synthases (TPSs), cytochrome P450 monooxygenases (P450s), and 2-oxoglutarate-dependent dioxygenases (ODDs). First, GGPP is converted to *ent*-kaurene by two monofunctional TPSs, *ent*-copalyl diphosphate synthase (CPS) and *ent*-kaurene synthase (KS). Then *ent*-kaurene is converted to intermediate GA_12_ catalyzed by two P450s, *ent*-kaurene oxidase (KO), and *ent*-kaurenoic acid oxidase (KAO). Finally, inactive precursor GA_12_ is converted to bioactive GAs through a series of oxidation steps catalyzed by two ODDs, GA 20-oxidase (GA20ox), and GA 3-oxidase (GA3ox). Additionally, the conversion of bioactive GAs and their immediate precursors into inactive GAs is through 2β-hydroxylation catalyzed by another ODD enzyme GA 2-oxidase (GA2ox) [7,8].

The genus *Anoectochilus* (Orchidaceae) is a perennial herb, which comprises more than 40 species that are widespread throughout tropical regions. Several species of this genus have been used in Chinese folk medicines, such as *A. roxburghii* (Wall.) Lindl., *A. formosanus* Hayata, and *A. koshunensis* Hayata. Of these plants, *A. roxburghii* is distributed in southern China, Japan, Sri Lanka, India, and Nepal [9,10,11], is also called “King Medicine” because of its diverse pharmacological effects such as the treatment of hypertension, fever, liver, and lung disease [12]. Due to its ornamental value and medicinal properties, the constantly increasing demand for wild *A. roxburghii* has resulted in its overexploitation and depletion. Thus far, seed propagation is regarded as the most efficient way to propagate native terrestrial orchids [13]. Orchid seed germination depends on mycorrhizal fungi because of lacking of reserve in minute seeds under natural conditions [14]. *In vitro*, symbiotic seed germination can be a cumbersome process, there is need to isolate symbiotic fungi from collected roots and the isolated fungi must then be identified and screened for germination-promoting strains. Asymbiotic seed germination can be a more straightforward process because it does not need to isolate mycobionts, which can germinate seeds of orchid taxa [15].

Zhao *et al.* [16] compared gene expression in symbiotically germinated and ungerminated seeds of the epiphytic orchid species *Dendrobium officinale* and revealed putative genes involved in symbiotic seed germination. Perotto *et al.* [17] used 454 pyrosequencing to generate a substantial sequence dataset of genes expressed in mycorrhizal protocorms (*Serapias vomeracea* protocorm and *Tulasnella calospora*), and revealed a symbiotic rather than an antagonistic plant-fungus relationship in mycorrhizal orchid protocorms. These studies have provided new insight into the process of symbiotic germination of orchid seeds. However, the molecular mechanisms of orchid seed germination are still poorly understood. In the present study, we comparatively analyzed gene expression between asymbiotic and symbiotic germination of *A. roxburghii* seeds using RNA-Seq technology, and tried to understand the seed germination process by focus on the expression profiles of genes involved in GA-GID1-DELLA regulatory module. The resultant transcriptome data and analysis results are very useful resources for gene discovery, and will be helpful to understand the molecular mechanism of orchid seed germination and the symbiotic orchid-fungus relationship at the germination stage.

## 2. Results

### 2.1. Transcriptome Profile of A. roxburghii Dry and Germinating Seeds

**Figure 1:**
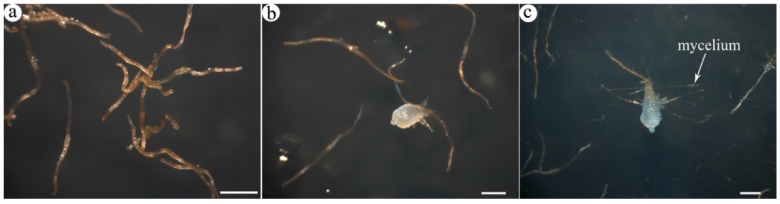
Light micrographs of germinating seeds of *A. roxburghii*: (**a**) DS, dry seeds; (**b**) AGS, asymbiotic germinating seeds; (**c**) SGS, symbiotic germinating seeds; (**b**,**c**) the third-stage seeds, appearance of promeristem. Scale bars = 0.5 mm.

**Figure 2:**
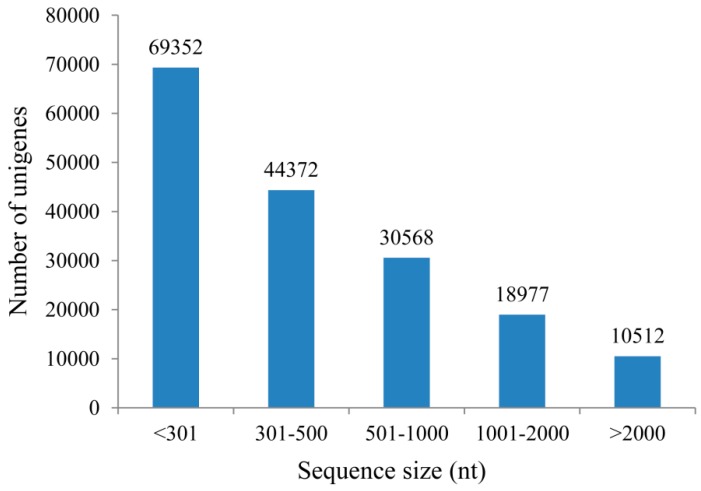
Length distribution of Unigene.

Total RNAs were isolated from DS, AGS and SGS (Figure 1), respectively. 3 μg of total RNA with high quality was equally pooled from each sample for sequencing library construction and they were subjected to Illumina sequencing by the HiSeq 4000 platform. In this study, all samples were sequenced in two biological replicates which were found to be highly reproducible (Figure S1). A total of 53,140,236–56,491,050 raw reads with the length of 150 bp were generated and 41,747,128–48,108,916 clean reads were obtained (Table 1). The raw reads are available at the NCBI SRA database under the accession number PRJNA299493. The cleaned reads were used to assemble the transcripts and a total of 245,063 transcripts were generated with a mean length of 793 nt, an N50 of 1389 nt. Among the transcripts representing the same gene, the longest one was considered as unigene. Finally, we obtained 173,781 unigenes with a mean length of 653 nt and an N50 of 1060 nt. The range in unigene length was from 201 to 16,676 nt, and the length distribution of unigene is illustrated in Figure 2.

**Table 1:** Output statistics of sequencing.

Sample	Raw Reads	Clean Reads	Error (%)	Q20 (%)	Q30 (%)	GC Content (%)
DS_1 ^1^	56,491,050	48,108,916	0.03	94.13	86.82	51.13
DS_2 ^1^	53,963,022	45,099,394	0.03	94.22	87.19	51.27
AGS_1 ^2^	54,662,424	41,747,128	0.02	95.59	89.26	47.85
AGS_2 ^2^	54,531,822	42,560,466	0.03	94.79	87.91	48.06
SGS_1 ^3^	53,273,636	43,590,926	0.03	93.96	86.59	48.74
SGS_2 ^3^	53,140,236	44,182,242	0.03	94.31	87.24	48.96

^1^ DS_1&DS_2: two biological replicates; ^2^ AGS_1&AGS_2: two biological replicates; ^3^ SGS1&SGS2: two biological replicates.

### 2.2. Unigene Functional Annotation

**Figure 3:**
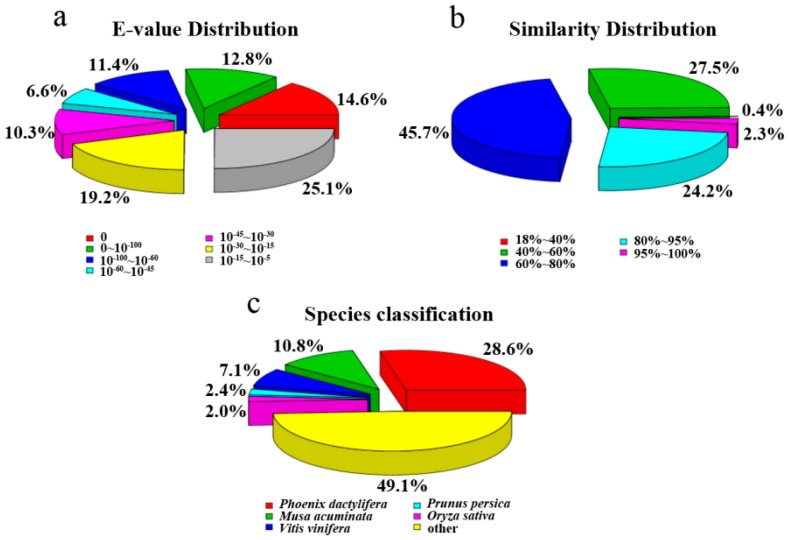
Characteristics of similarity search of unigenes against NR database: (**a**) *E*-value distribution of BLAST hits for each unigene with a cutoff *E*-value of 10^−5^; (**b**) Similarity distribution of the top BLAST hit for each unigene; (**c**) Species distribution of the top BLAST hit for each unigene in the NR database.

For assignments of the unigene, a total of 51,514 (29.64%) unigenes with significant BLAST hits were returned. Among them, 39,262 (22.59%), 13,468 (7.74%), 16,815 (9.67%), 28,414 (16.35%), 33,702 (19.39%), 33,960 (19.54%), and 12,494 (7.18%) unigenes had hits in the Nr, Nt, KOG, Swiss-Prot, Pfam, GO, and KEGG database, respectively. The *E*-value and similarity distributions of the top hits in the Nr database analysis revealed that 45.4% (17,825) and 26.5% (10,404) of genes showed significant homology (*E*-value < 10^−45^) or high similarity (greater than 80%), respectively (Figure 3a,b). Based on Nr annotation, approximately 50.9% of annotated unigenes were assigned with a best score to the sequence from the top five species, *i.e.*, *Phoenix dactylifera* (28.6%), *Musa acuminata* (10.8%), *Vitis vinifera* (7.1%), *Prunus persica* (2.4%), *Oryza sativa* (2.0%) (Figure 3c).

**Table 2:** Unigenes involved in GA-GID1-DELLA regulatory module.

Pathway	Gene Name	Number of Unigenes in Transcriptome
GA biosynthesis	*ent*-Copalyl diphosphate synthase (CPS)	2
	*ent*-Kaurene synthase (KS)	1
	*ent*-Kaurene oxidase (KO)	2
	*ent*-Kaurenoic acid oxidase (KAO)	1
	GA20-oxidase (GA20ox)	9
	GA3 beta-hydroxylase (GA3ox)	5
GA catabolism	GA2-oxidase (GA2ox)	11
GA signaling downstream	GA receptor GID1	5
	DELLA family protein	11
	F-box protein GID2	2

### 2.3. The Unigenes Encoding the Proteins Involved in GA-GID1-DELLA Regulatory Module

GA-GID1-DELLA signaling module in plants is shaped by phytohormone GA, receptor GID1 and repressor DELLA, and it plays a pivotal role in regulating seed germination [18,19]. Therefore, we searched the unigenes encoding proteins involved in GA-GID1-DELLA module by searching the annotation with gene name as key words. As a result, 20 unigenes involved in GA biosynthesis pathway were found, including two unigenes encoding *ent*-copalyl diphosphatesynthase (CPS) and one unigene encoding *ent*-kaurene synthase (KS), both which convert precursor *trans*-geranylgeranyl diphosphate (GGPP) to *ent*-kaurene [20], two unigenes encoding *ent*-kaurene oxidase (KO), and one unigene encoding *ent*-kaurenoic acid oxidase (KAO) which covert *ent*-kaurene to intermediate GA_12_, nine unigenes encoding GA 20-oxidase (GA20ox), and five unigenes encoding GA 3-oxidase (GA3ox) which covert inactive GA_12_ to bioactive GAs. Eleven unigenes encoding GA 2-oxidase (GA2ox) which catalyze the conversion of bioactive GAs to inactive catabolites in GA catabolic pathway were found [8,21]. Moreover, the unigenes associated with GA signaling, for instance, 5 unigenes encoding GA receptor GIBBERELLIN-INSENSITIVE DWARF1 (GID1), 11 unigenes for repressor DELLA, and 2 unigenes for F-box protein GID2 were found (Table 2, Tables S1 and S2).

### 2.4. Differential Expression Genes at Seed Germination with or without Mycorrhiza Fungi

**Figure 4:**
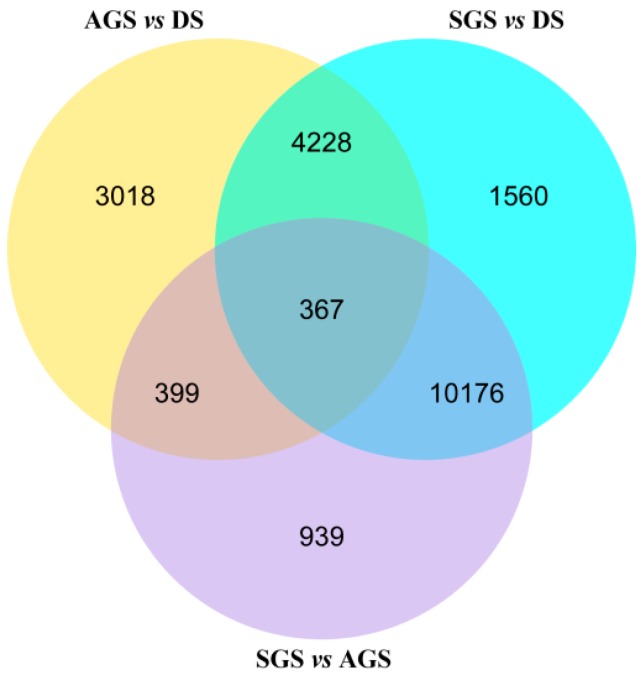
Venn diagram of differentially expressed genes in the germination *of A. roxburghii* seeds. “X *vs.* Y” means Y is control.

**Figure 5:**
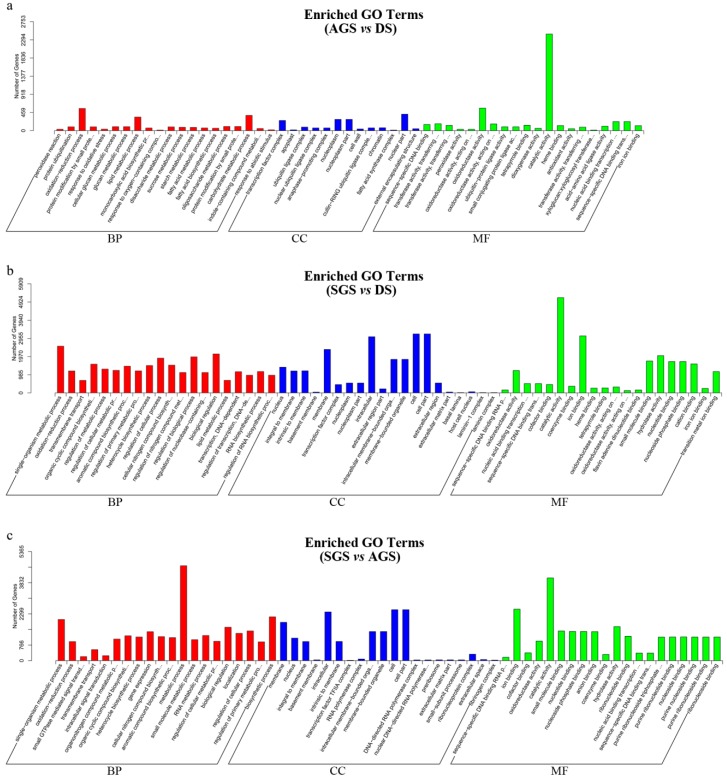
GO functional classification of differentially expressed genes. (**a**,**b**) Third-stage seeds from asymbiotic germination (AGS) or the same stage seeds from symbiotic germination (SGS), both compared with dry seeds (DS), respectively; Also, (**c**) SGS compared with AGS. BP, Biological Processes, CC, Cellular Components, and MF, Molecular Function.

In order to obtain the differential expression genes (DEGs), the gene expression profiles of AGS and SGS were compared to those of DS, respectively. Meanwhile, the gene expression profiles of SGS was compared to those of AGS. A total of 8012, 16,331, and 11,881 DEGs were observed in AGS *vs.* DS, SGS *vs.* DS, and SGS *vs.* AGS, respectively (Figure 4 and Figure S2). The venn diagram showed that a total of 3018, 1560, and 939 DEGs were only changed in AGS *vs.* DS, SGS *vs.* DS, and SGS *vs.* AGS, respectively (Figure 4). In order to analyze their functions, these DEGs were annotated in GO. Under the GO classification, the unigenes involved in biological process, cellular component and molecular function in SGS *vs*. DS were very different from those of AGS *vs.* DS, but interestingly, the number of unigenes associated with catalytic activity is very high in both (Figure 5A,B), indicating that these unigenes annotated to catalytic activity are important for *A. roxburghii* seed germination. For the GO classification of DEGs in SGS *vs*. AGS, the number of unigenes associated with metabolic process and catalytic activity are very high (Figure 5C), implying that mycorrhizal fungi might influence the metabolic process by playing a role in regulating catalytic activity. When DEGs were searched against KEGG pathway, 3057 (AGS *vs.* DS), 7964 (SGS *vs.* DS), and 6586 (SGS *vs.* AGS) DEGs with significant hits were returned, respectively. Among these pathways, the top 20 pathways for the three groups of DEGs were summarized (Table 3), and it is noteworthy that “ubiquitin mediated proteolysis” is listed in SGS *vs.* DS and SGS *vs.* AGS, but it is not in AGS *vs.* DS. It is well known that the DELLA proteins are polyubiquitinated by SCF^SLY1/GID2^ E3 ubiquitin ligase and then proteolyzed via the 26S proteasome [22], and regulate arbuscule formation in arbuscular mycorrhizal symbiosis [23], so the results suggested that DELLA proteins also may be required in orchid symbiosis and fungi may influence on GA-GID1-DELLA module by mediating DELLA proteolysis.

**Table 3:** Summary of top twenty KEGG pathway in DEGs.

**AGS *vs.* DS**			
**#**	**KEGG Pathway**	**Number of Unigenes**	**Percentage (%)**
1	Phenylpropanoid biosynthesis	89	2.91
2	Starch and sucrose metabolism	73	2.39
3	Carbon metabolism	71	2.32
4	Plant hormone signal transduction	68	2.22
5	Biosynthesis of amino acids	68	2.22
6	Plant-pathogen interaction	62	2.03
7	Phenylalanine metabolism	53	1.73
8	Glycolysis/Gluconeogenesis	39	1.28
9	Protein processing in endoplasmic reticulum	33	1.08
10	Cyanoamino acid metabolism	32	1.05
11	Endocytosis	32	1.05
12	Glycerophospholipid metabolism	30	0.98
13	Glyoxylate and dicarboxylate metabolism	29	0.95
14	Photosynthesis	27	0.88
15	Amino sugar and nucleotide sugar metabolism	27	0.88
16	Purine metabolism	27	0.88
17	Flavonoid biosynthesis	26	0.85
18	Carbon fixation in photosynthetic organisms	24	0.79
19	Glutathione metabolism	23	0.75
20	Pyruvate metabolism	23	0.75
	**SGS *vs.* DS**		
**#**	**KEGG Pathway**	**Number of Unigenes**	**Percentage (%)**
1	Biosynthesis of amino acids	135	1.70
2	Carbon metabolism	134	1.68
3	Starch and sucrose metabolism	112	1.41
4	Ribosome	107	1.34
5	Phenylpropanoid biosynthesis	105	1.32
6	Spliceosome	98	1.23
7	Purine metabolism	87	1.09
8	Protein processing in endoplasmic reticulum	87	1.09
9	RNA transport	83	1.04
10	Amino sugar and nucleotide sugar metabolism	71	0.89
11	Plant-pathogen interaction	71	0.89
12	Glycolysis / Gluconeogenesis	70	0.88
13	Ubiquitin mediated proteolysis	66	0.83
14	Pyrimidine metabolism	66	0.83
15	Endocytosis	66	0.83
16	Oxidative phosphorylation	63	0.79
17	Phenylalanine metabolism	62	0.78
18	Cell cycle	57	0.72
19	Peroxisome	56	0.70
20	Pyruvate metabolism	55	0.69
**SGS *vs.* AGS**			
**#**	**KEGG Pathway**	**Number of Unigenes**	**Percentage (%)**
1	Biosynthesis of amino acids	111	1.69
2	Ribosome	103	1.56
3	Carbon metabolism	97	1.47
4	Spliceosome	96	1.46
5	Protein processing in endoplasmic reticulum	76	1.15
6	RNA transport	75	1.14
7	Purine metabolism	72	1.09
8	Starch and sucrose metabolism	70	1.06
9	Oxidative phosphorylation	64	0.97
10	Endocytosis	60	0.91
11	Ubiquitin mediated proteolysis	57	0.87
12	Pyrimidine metabolism	55	0.84
13	Phenylpropanoid biosynthesis	53	0.80
14	Ribosome biogenesis in eukaryotes	51	0.77
15	Amino sugar and nucleotide sugar metabolism	50	0.76
16	Cell cycle	50	0.76
17	mRNA surveillance pathway	46	0.70
18	RNA degradation	45	0.68
19	Glycolysis/Gluconeogenesis	45	0.68
20	Peroxisome	43	0.65

### 2.5. DEGs Involved in GA-GID1-DELLA Regulatory Module

Further analysis of DEGs between symbiotic and asymbiotic seed germination (SGS *vs.* AGS), six genes involved in GA-GID1-DELLA regulatory module were identified, including two genes encoding GA20ox, two genes encoding GA2ox, and two genes encoding DELLA protein (Table 4). The results suggest that fungi mediate the GA-GID1-DELLA regulatory module through changing in expression of genes encoding GA20ox, GA2ox and DELLA protein.

**Table 4:** DEGs (SGS *vs.* AGS ^1^) involved in GA-GID1-DELLA regulatory module.

Pathway	Gene Name	Number	Unigene ID	log_2_fold ^2^	padj ^3^
GA biosynthesis	*ent*-Copalyl diphosphate synthase (CPS)	0	–	–	–
	*ent*-Kaurene synthase (KS)	0	–	–	–
	*ent*-Kaurene oxidase (KO)	0	–	–	–
	*ent*-Kaurenoic acid oxidase (KAO)	0	–	–	–
	GA20-oxidase (GA20ox)	2	c99861_g1	2.17	1.97 × 10^−3^
			c90765_g1	4.30	8.15 × 10^−4^
	GA3 beta-hydroxylase (GA3ox)	0	–	–	–
GA catabolism	GA2-oxidase (GA2ox)	2	c99070_g1	2.98	3.03 × 10^−7^
			c106997_g1	4.98	5.18 × 10^−5^
GA signaling	GA receptor GID1	0	–	–	–
	DELLA family protein SLR1	2	c85242_g1	1.62	1.51 × 10^−2^
			c93049_g1	1.83	3.58 × 10^−3^
	F-box protein GID2	0	–	–	–

^1^ SGS *vs* AGS: the gene expression profiles of SGS was compared to those of AGS; ^2^ log2fold: fold changes of the six DEGs were logarithmic transformed which were determined by RNA-seq; ^3^ padj: adjusted *p*-value.

## 3. Discussion

### 3.1. The Complexity of Orchid Seed Germination

Seed germination is a comprehensive process. After absorbing water, the mature seed quickly shifts from a program of maturation to germination-driven and then prepares for seedling growth [24]. Almost all orchid seeds are extremely small (0.3–14 μg), dust-like, and contain notably few nutrient reserves [16]. Further, in the majority of orchid species, seed germination and early seedling development is largely dependent on mycorrhizal fungi under natural conditions because of the lack of nutritional reserves [25,26], thus the process of orchid seed germination in nature is complex and special, involving various processes such as colonization, plant growth stimulation, plant response to fungi, *etc.* According to the GO classification of DEGs (Figure 4A,B), We can clearly see that DEGs from AGS *vs.* DS and SGS *vs.* DS involved in many biological processes, cellular components, and molecular functions, and the result also suggested that orchid seed germination is a complex, multi-stage process and requires the coordinated expression of numerous different functional genes. In addition, the DEGs were more in SGS *vs.* DS than in AGS *vs.* DS, implying that symbiotic fungi promote metabolic process in *A. roxburghii* seed germination and it is also a reflection of the fact that orchid seed germination under natural condition is a very complex physiological and biochemical process.

### 3.2. The Role of Gibberellins in Seed Germination and Unigenes Related to the GA Signaling Pathway

GA and ABA play significant but antagonistic roles in the regulation of the seed germination process. Whereas GA promotes seed germination, ABA inhibits the process. Furthermore, the antagonistic relationship and the ratio between these two hormones regulate the germination process [27]. The GA-deficient *Arabidopsis thaliana* mutant *ga1–3* is defective in seed germination because of containing greatly reduced levels of bioactive GAs [28,29]. In recent years, major progress has been made in understanding the molecular mechanism of GA action, that is GA-GID1-DELLA. In brief, a soluble receptor GID1 or GID1-like elevate their direct interaction with DELLA proteins in the presence of bioactive GAs, the GA-GID1-DELLA complex is recognized by the GA-specific F-box proteins (GID2 in *Oryza sativa* and SLY1 in *A*. *thaliana*) for ubiquitination of the DELLA protein, which is then degraded through the 26S proteasome pathway. The degradation of DELLAs will relieve the DELLA-mediated growth restraint [30,31,32]. In this study, 49 unigenes involved in the GA-GID1-DELLA regulatory module were identified (Table 2), and the number of unigenes identified is similar to that obtained from lettuce seed (43 unigenes involved in GA-GID1-DELLA module), which implies that the assembled transcriptome is a reliable one and might be a very useful resource for gene annotation and discovery. Among the 49 gene set, there are two unigenes with better similarity to the rice *GID2* than the Arabidopsis *SLY1*. This is not surprising since orchids are monocotyledonous species and thus more closely related to rice than Arabidopsis. GA can promote seed germination in opposition to ABA by enhancing the proteasome-dependent degradation of repressor DELLA and many genes involved in the regulation of the GA signaling pathway, which mainly divided into three categories: genes involved in GA biosynthesis, catabolism, and GA signaling downstream (receptor and repressor).

### 3.3. Symbiotic Fungi Influence on the GA-GID1-DELLA Regulatory Module

The GA-GID1-DELLA growth regulatory mechanism enables a flexible response to environmental variability, such as light, high salinity, temperatures, and pathogens [33]. Symbiotic fungi as a biotic factor have an effect on the GA-GID1-DELLA regulatory module. The gene expression profiles of SGS were compared to those of AGS, six DEGs involved in GA-GID1-DELLA module were identified (Table 4). Of the six DEGs, two unigenes encoded a GA20ox, two unigenes encoded a GA2ox, and two unigenes encoded a DELLA protein. It is widely accepted, and there is abundant evidence that GA20ox, GA3ox, and GA2ox catalyze key regulatory steps in GA biosynthesis [20], GA20ox, and GA3ox that catalyze the final steps in the synthesis of bioactive GAs. However, GA2ox converts bioactive GA or their precursors to inactive forms by 2β-hydroxylation [4,34,35]. Our results also proved this point and suggested that fungi have effect on GA biosynthesis through the modulation of the expression of members of two small families, *GA20ox* and *GA2ox*.

DELLA proteins function as repressors in GA signaling and are highly conserved in *Arabidopsis*, and in several crop plants, including barley, grape, maize, rice, and wheat. A single DELLA protein gene *SLENDER RICE1* (*SLR1*) and *SLENDER1* (*SLN1*) is present in rice and barley, respectively. Surprisingly, five DELLA protein genes (*GA-Insensitive* (*GAI*), Repressor of *ga1–3* (*RGA*), *RGA-like1* (*RGL1*), *RGL2,* and *RGL3*) have been identified in *Arabidopsis*, with RGL2 playing a major role in GA-dependent germination of *Arabidopsis* seeds. [36,37]. Three independent studies have now shown that DELLA proteins are required for AM development [23,38,39]. The results illustrate two DEGs (from SGS *vs.* AGS) encoding DELLA protein SLR1 and suggested DELLA proteins play an important role in orchid symbiotic seed germination, meanwhile, are also required for orchid-fungus symbiosis formation. All in all, symbiotic fungi might have effect on GA-GID1-DELLA regulatory module only through the modulation of the expression of *GA20ox*, *GA2ox*, and *DELLA*, but the specific mechanism still remains undefined.

## 4. Materials and Methods

### 4.1. Symbiotic and Asymbiotic Germination of Anoectochilus roxburghii Seeds

Mature, undehiscent *A. roxburghii* capsules were collected from Yongchun county in Fujian province, southeast China. The capsules were surface sterilized in 75% ethanol for 1 min and 2.5% NaClO for 5 min, then rinsed three times in sterile water, followed by transferring to sterile filter paper for drying and were split longitudinally with a sterile scalpel. Lastly, axenic seeds were cleaned from the capsule debris and stored in a 1.5-mL centrifuge tube on silica gel at 4 °C. *A. roxburghii* seeds were identified by DNA barcoding [40,41,42].

Co-inoculation of mycorrhizal fungi and *A. roxburghii* seeds was performed in a 9 cm petri dish. Sowing the axenic seeds onto a square of autoclaved nylon cloth (4 cm × 4 cm) by an autoclaved writing brush, and the nylon cloth was positioned on water agar medium (1.2% agar). An inoculum of isolate Ar-34 (unpublished data), obtained from *A. roxburghii* and consisting of four 3 mm × 3 mm portions of actively growing mycelium, was placed in each petri dish. Asymbiotic seed germination was performed on 1/2MS culture media. All treatments were maintained at 25 °C with a 12 h/12 h light-dark cycle. Dry and the third-stage seeds (Figure 1) were collected, immediately frozen in liquid nitrogen, and stored at −80 °C prior to RNA extraction.

### 4.2. Total RNA Extraction

Total RNAs were extracted from each sample using RNeasy Plant Mini Kit (QIAGEN, Hilden, Germany) and treated with an RNase-free DNase I digestion kit (Aidlab, Beijing, China) to remove residual genomic DNA. RNA degradation and contamination was monitored on 1% agarose gels. RNA purity was checked using the NanoPhotometer^®^ spectrophotometer (IMPLEN, Westlake Village, CA, USA). RNA was quantified using Qubit^®^ 2.0 Flurometer (Life Technologies, San Diego, CA, USA), checked for integrity on an Agilent 2100 Bioanalyzer (Agilent Technologies, Palo Alto, CA, USA). Finally, 3 μg of total RNA with RIN (RNA integrity number) above 7.0 was equally pooled from each sample for cDNA library construction, and each sample has two biological replicates.

### 4.3. cDNA Library Construction and Sequencing

Library construction, quality detection and sequencing were performed by the Novogene Bioinformatics Institute (Beijing, China). Sequencing libraries were generated using NEBNext^®^ Ultra^™^ RNA Library Prep Kit (NEB, Ipswich, MA, USA) and the index-coded samples were clustered using TruSeq PE Cluster Kit v3-cBot-HS (Illumia, San Diego, CA, USA), according to the manufacturer’s instructions. After cluster generation, the library preparations were sequenced on an Illumina Hiseq 4000 instrument with 150 bp paired-end reads.

### 4.4. Data Filtering and de Novo Assembly

Following cDNA library sequencing, sequencing-received raw image data were transformed by base calling into sequence data, after quality filtering, raw reads (sequence data without fungi reads) were obtained. High-quality clean reads were generated from the raw reads of each library by removing adaptor sequences, duplicated sequences, reads with unknown nucleotides (“N”) larger than 10%, and low-quality reads with more than 50% of quality value ≤20 bases. All the downstream analyses were based on the clean reads.

De novo assembly was applied to construct transcripts from the clean reads because of the absence of reference genomic sequences, and transcriptome assembly was accomplished using Trinity [43] with min_kmer_cov set to 2 by default. The longest transcript from each gene were designated unigenes.

### 4.5. Functional Annotation and Metabolic Pathway Analysis of Unigenes

The unigenes were searched against four databases, NCBI [44] non-redundant protein database (Nr), NCBI nucleotide database (Nt), as well as SwissProt protein database [45] with *E* value less than 10^−5^, and euKaryotic Ortholog Groups of proteins (KOG) database [46] with *E* value threshold of 10^−3^ by BLASTX (version 2.2.28+, NCBI, Bethesda, MA, USA). And the protein domains were predicted by HMMER 3.0 package in protein family (Pfam) database [47]. With Nr and Pfam annotation, the unigenes were annotated according to biological process, cellular component, and molecular function by Blast2GO v2.5 in Gene Ontology (GO) database [48]. Metabolic pathway analysis was performed using the Kyoto Encyclopedia of Genes and Genomes Pathway database (KEGG) [49] and related software KAAS (KEGG Automatic Annotation Server, Tokyo, Japan).

### 4.6. Analysis of Different Expression Genes

Dry seeds (DS) and the third-stage seeds from asymbiotic germination (AGS) or symbiotic germination (SGS) were used as materials to investigate different expression genes by digital gene expression profile analysis. Clean reads were mapped back onto the assembled transcriptome by RSEM (v1.2.15) software [50] with bowtie2 (mismatch 0), and readcount for each gene was obtained from the mapping results. Next, differential expression analysis was performed using the DESeq R package (v1.10.1) [51]. The resulting *p* values were adjusted by the method of Benjamini and Hochberg [52] and adjusted *p*-value of 0.05 was set as the threshold for selecting genes that differentially expressed.

## 5. Conclusions

A better understanding of germination and the role for mycobiont in germination is needed for conservation of orchid populations. Due to the obligate association with a mycobiont, the germination of orchid seeds is extremely complex and varied [53]. It is a multi-stage process requiring numerous genes coordinately regulated both spatially and temporally [54].

In this study, we generated the first large-scale transcriptome dataset of the *A. roxburghii* seed that examined both symbiotic and asymbiotic seed germination. We identified the functions and metabolic pathways of genes expressed in the *A. roxburghii* seed, and focused on genes involved in the GA-GID1-DELLA regulatory module. In total, we identified 49 genes related to the regulatory module, of which six genes were differential expressed in SGS *vs.* AGS. The generated transcriptome dataset of *A. roxburghii* seed lays the foundation for further studies of the molecular mechanisms related to orchid seed germination and orchid-fungus symbiosis. Furthermore, the focus on expression profile of genes involved in the GA-GID1-DELLA module provides new insights into the molecular mechanisms of *A. roxburghii* seed germination.

## Supplementary Materials

Supplementary materials can be found at http://www.mdpi.com/1422-0067/16/12/26224/s1.

## Abbreviations

ABA: abscisic acid
AM: arbuscular mycorrhiza
AGS: asymbiotic germinating seeds
CPS: *ent*-copalyl diphosphate synthase
DEGs: differentially expressed genes
DS: dry seeds
GA: gibberellin
GA20ox: gibberellin 20-oxidase
GA3ox: gibberellin 3-oxidase
GA2ox: gibberellin 2-oxidase
GGPP: *trans*-geranylgeranyl diphosphate
GID1: GIBBERELLIN INSENSITIVE DWARF1
GO: Gene Ontology
KAO: *ent*-kaurenoic acid oxidase
KEGG: the Kyoto Encyclopedia of Genes and Genomes dababase
KO: *ent*-kaurene oxidase
KOG: euKaryotic Ortholog Groups database
KS: *ent*-kaurene synthase
Nr: NCBI non-redundant protein sequences database
Nt: NCBI nucleotide database
ODDs: 2-oxoglutarate-dependent dioxygenases
Pfam: protein family database
RIN: RNA integrity numbers
SGS: symbiotic germinating seeds
TPSs: terpene synthases

## References

[1] Jiang C., Fu X. (2007). GA action: Turning on de-DELLA repressing signaling. Curr. Opin. Plant Biol..

[2] Sun T.-P., Gubler F. (2004). Molecular mechanism of gibberellin signaling in plant. Annu. Rev. Plant Biol..

[3] Voegele A., Linkies A., Müller K., Leubner-Metzger G. (2011). Members of the gibberellin receptor gene family GID1 (GIBBERELLIN INSENSITIVE DWARF1) play distinct roles during *Lepidium. sativum* and *Arabidopsis thaliana* seed germination. J. Exp. Bot..

[4] Achard P., Genschik P. (2009). Releasing the brakes of plant growth: How GAs shutdown DELLA proteins. J. Exp. Bot..

[5] Sun T.-P. (2011). The molecular mechanism and evolution of the GA–GID1–DELLA signaling module in plants. Curr. Biol..

[6] Bari R., Jones J.D.G. (2009). Role of plant hormones in plant defence responses. Plant Mol. Biol..

[7] Yamaguchi S. (2008). Gibberellin metabolism and its regulation. Annu. Rev. Plant Biol..

[8] Wang Y.-J., Deng D.-X. (2014). Molecular basis and evolutionary pattern of GA–GID1–DELLA regulatory module. Mol. Genet. Genom..

[9] (1999). Delectis Florae Reipublicae Popularis Sinicae, Agendae Academiae Sinicae Edita. Flora Reipublicae Popularis Sinicae.

[10] He C.-N., Wang C.-L., Guo S.-X., Yang J.-S., Xiao P.-G. (2006). A novel flavonoid glucoside from *Anoectochilus* roxburghii (Wall.) Lindl. J. Integr. Plant Biol..

[11] Gutiérrez R.M.P. (2010). Orchids: A review of uses in traditional medicine, its phytochemistry and pharmacology. J. Med. Plant Res..

[12] Fujian Institute of Traditional Chinese Medicine (1982). Record of Fujian Materia Medica.

[13] Stewart S.L., Kane M.E. (2006). Asymbiotic seed germination and *in vitro* seedling development of *Habenaria. macroceratitis* (Orchidaceae), a rare Florida terrestrial orchid. Plant Cell Tissue Organ.

[14] Arditti J. (1967). Factors affecting the germination of orchid seeds. Bot. Rev..

[15] Johnson T.R., Stewart S.L., Dutra D., Kane M.E., Richardson L. (2007). Asymbiotic and symbiotic seed germination of *Eulophia. alta* (Orchidaceae)—Preliminary evidence for the symbiotic culture advantage. Plant Cell Tissue Organ.

[16] Zhao M.-M., Zhang G., Zhang D.-W., Hsiao Y.-Y., Guo S.-X. (2013). ESTs analysis reveals putative genes involved in symbiotic seed germination in *Dendrobium officinale*. PLoS ONE.

[17] Perotto S., Rodda M., Benetti A., Sillo F., Ercole E., Rodda M., Girlanda M., Murat C., Balestrini R. (2014). Gene expression in mycorrhizal orchid protocorms suggests a friendly plant–fungus relationship. Planta.

[18] Tyler L., Thomas S.G., Hu J., Dill A., Alonso J.M., Ecker J.R., Sun T.-P. (2004). DELLA proteins and gibberellin-regulated seed germination and floral development in *Arabidopsis*. Plant Physiol..

[19] Seo M., Nambara E., Choi G., Yamaguchi S. (2009). Interaction of light and hormone signals in germinating seeds. Plant Mol. Biol..

[20] Hedden P., Phillips A.L. (2000). Gibberellin metabolism: New insights revealed by the genes. Trends Plant Sci..

[21] Hu J., Mitchum M.G., Barnaby N., Ayele B.T., Ogawa M., Nam E., Lai W.-C., Hanada A., Alonso J.M., Ecker J.R. (2008). Potential sites of bioactive gibberellin production during reproductive growth in Arabidopsis. Plant Cell.

[22] Murase K., Hirano Y., Sun T.P., Hakoshima T. (2008). Gibberellin-induced DELLA recognition by the gibberellin receptor GID1. Nature.

[23] Floss D.S., Levy J.G., Lévesque-Tremblay V., Pumplin N., Harrison M.J. (2013). DELLA proteins regulate arbuscule formation in arbuscular mycorrhizal symbiosis. Proc. Natl. Acad. Sci. USA.

[24] Rajjou L., Duval M., Gallardo K., Catusse J., Bally J., Job C., Job D. (2012). Seed germination and vigor. Annu. Rev. Plant Biol..

[25] Rasmussen H.N. (1995). Terrestrial Orchids: From Seed to Mycotrophic Plant.

[26] Zettler L.W., Hofer C.J. (1998). Propagation of the little club-spur orchid (*Platanthera. clavellata*) by symbiotic seed germination and its ecological implications. Environ. Exp. Bot..

[27] Weiss D., Ori N. (2007). Mechanisms of cross talk between gibberellin and other hormones. Plant Physiol..

[28] Sun T.-P., Kamiya Y. (1994). The Arabidopsis GA1 locus encodes the cyclase *ent*-kaurene synthetase A of gibberellin biosynthesis. Plant Cell.

[29] Cao D., Cheng H., Wu W., Soo H.M., Peng J. (2006). Gibberellin mobilizes distinct DELLA-dependent transcriptomes to regulate seed germination and floral development in *Arabidopsis*. Plant Physiol..

[30] Fu X., Richards D.E., Fleck B., Xie D., Burton N., Harberd N.P. (2004). The *Arabidopsis* mutant sleepy1^gar2−1^ protein promotes plant growth by increasing the affinity of the SCF^SLY1^ E3 ubiquitin ligase for DELLA protein substrates. Plant Cell.

[31] Itoh H., Ueguchi-Tanaka M., Sato Y., Ashikari M., Matsuoka M. (2002). The gibberellin signaling pathway is regulated by the appearance and disappearance of SLENDER RICE1 in Nuclei. Plant Cell.

[32] Hussain A., Cao D.-N., Cheng H., Wen Z.-L., Peng J.-R. (2005). Identification of the conserved serine/threonine residues important for gibberellin-sensitivity of *Arabidopsis* RGL2 protein. Plant J..

[33] Harberd N.P., Belfield E., Yasumura Y. (2009). The angiosperm gibberellin-GID1-DELLA growth regulatory mechanism: how an “inhibitor of an inhibitor” enables flexible response to fluctuating environments. Plant Cell.

[34] Schomburg F.M., Bizzell C.M., Lee D.J., Zeevaart J.A., Amasino R.M. (2003). Overexpression of a novel class of gibberellin 2-oxidases decreases gibberellin levels and creates dwarf plants. Plant Cell.

[35] Thomas S.G., Phillips A.L., Hedden P. (1999). Molecular cloning and functional expression of gibberellin 2-oxidases, multifunctional enzymes involved in gibberellin deactivation. Proc. Natl. Acad. Sci. USA.

[36] Toh S., Imamura A., Watanabe A., Nakabayashi K., Okamoto M., Jikumaru J., Hanada A., Aso Y., Ishiyama K., Tamura N. (2008). High temperature-induced abscisic acid biosynthesis and its role in the inhibition of gibberellin action in *Arabidopsis* seeds. Plant Physiol..

[37] Fleet C.M., Sun T.-P. (2005). A DELLAcate balance: The role of gibberellin in plant morphogenesis. Curr. Opin. Plant Biol..

[38] Gutjahr C. (2014). Phytohormone signaling in arbuscular mycorhiza development. Curr. Opin. Plant Biol..

[39] Foo E., Ross J.J., Jones W.T., Reid J.B. (2013). Plant hormones in arbuscular mycorrhizal symbioses: An emerging role for gibberellins. Ann. Bot..

[40] Gismondi A., Rolfo M.F., Leonardi D., Rickards O., Canini A. (2012). Identification of ancient *Olea europaea* L. and *Cornus. mas* L. by DNA barcoding. Cr. Biol..

[41] Gismondi A., Fanali F., Martínez Labarga J.M., Grilli Caiola M., Canini A. (2013). *Crocus sativus* L. genomics and different DNA barcode applications. Plant Syst. Evol..

[42] Gismondi A., di Marco G., Delorenzo M., Canini A. (2015). Upgrade of *Castanea. sativa* (Mill.) genetic resources by sequencing of barcode markers. J. Genet..

[43] Grabherr M.G., Haas B.J., Yassour M., Levin J.Z., Thompson D.A., Amit I., Adiconis X., Fan L., Raychowdhury R., Zeng Q. (2011). Full-length transcriptome assembly from RNA-Seq data without a reference genome. Nat. Biotechnol..

[44] NCBI. http://www.ncbi.nlm.nih.gov/.

[45] UniProt. http://www.ebi.ac.uk/uniprot/.

[46] KOG/COG. http://www.ncbi.nlm.nih.gov/COG/.

[47] Pfam. http://pfam.sanger.ac.uk/.

[48] Gene Ontology Consortium. http://geneontology.org/.

[49] KEGG. http://www.genome.jp/kegg/.

[50] Li B., Dewey C. (2011). RSEM: accurate transcript quantification from RNA-Seq data with or without a reference genome. BMC Bioinform..

[51] Anders S., Huber W. (2010). Differential expression analysis for sequence count data. Genome Biol..

[52] Benjamini Y., Hochberg Y. (1995). Controlling the false discovery rate: A practical and powerful approach to multiple testing. J. R. Stat. Soc. B.

[53] Rasmussen H.N., Dixon K.W., Jersáková J., Těšitelová T. (2015). Germination and seedling establishment in orchids: A complex of requirements. Ann. Bot..

[54] Potokina E., Sreenivasulu N., Altschmied L., Michalek W., Graner A. (2002). Differential gene expression during seed germination in barley (Hordeum vulgare L.). Funct. Integr. Genom..

